# Assessing the activity of nonsense-mediated mRNA decay in lung cancer

**DOI:** 10.1186/s12920-017-0292-z

**Published:** 2017-09-06

**Authors:** Meng Wang, Peiwei Zhang, Yufei Zhu, Xiangyin Kong, Zhenguo Zhang, Landian Hu

**Affiliations:** 1 State Key Laboratory of Medical Genomics, Institute of Health Sciences, Shanghai Institutes for Biological Sciences, Chinese Academy of Sciences and Ruijin Hospital, Shanghai Jiaotong University School of Medicine, Shanghai, People’s Republic of China; 2 Department of Biology, University of Rochester, Rochester, NY 14627 USA; 3 Present Address: 17062 Murphy Ave., Irvine, CA 92614 USA

**Keywords:** Lung adenocarcinoma, Cancer therapy, Tumor growth suppression, Nonsense-mediated mRNA decay

## Abstract

**Background:**

Inhibition of nonsense-mediated mRNA decay (NMD) in tumor cells can suppress tumor growth through expressing new antigens whose mRNAs otherwise are degraded by NMD. Thus NMD inhibition is a promising approach for developing cancer therapies. Apparently, the success of this approach relies on the basal NMD activity in cancer cells. If NMD is already strongly inhibited in tumors, the approach would not work. Therefore, it is crucial to assess NMD activity in cancers to forecast the efficacy of NMD-inhibition based therapy.

**Methods:**

Here we develop three metrics using RNA-seq data to measure NMD activity, and apply them to a dataset consisting of 72 lung cancer (adenocarcinoma) patients.

**Results:**

We show that these metrics have good correlations, and that the NMD activities in adenocarcinoma samples vary among patients: some cancerous samples show significantly stronger NMD activities than the normal tissues while some others show the opposite pattern. The variation of NMD activities among these samples may be partly explained by the varying expression of NMD effectors.

**Conclusions:**

In sum, NMD activity varies among lung cancerous samples, which forecasts varying efficacies of NMD-inhibition based therapy. The developed metrics can be further used in other cancer types to assess NMD activity.

**Electronic supplementary material:**

The online version of this article (10.1186/s12920-017-0292-z) contains supplementary material, which is available to authorized users.

## Background

Lung cancer is the leading cause of cancer-related deaths [1]: each year over a million of patients die of it, and millions of new cases are diagnosed. Such high incidence and mortality rates urge developing new effective treatments.

Recently, a new promising approach to treat cancers is to express new antigens in tumor cells through inhibiting nonsense-mediated mRNA decay (NMD) pathway [2, 3]. The mRNAs of these new antigens (owing to tumorous mutations) are normally degraded by NMD and thus invisible to immune systems. NMD is a cellular surveillance pathway for degrading mRNAs harboring premature termination codons (PTCs) [4]. When NMD is inhibited, these antigens can be expressed and trigger immune rejection of tumor cells. This method successfully suppressed tumor growth in mice implanted with a colon carcinoma cell line CT26 [2]. However, its efficacies in clinics and other types of tumors have not been evaluated so far. Particularly, the success of this method relies on the NMD activity of tumor cells. In another word, if tumor cells already have very low NMD activity, inhibiting NMD would not express substantial amount of new antigens and the method would not work. Therefore, it is necessary to evaluate NMD activities in different tumor types for potential application of NMD-inhibition-based therapies.

It has been reported that tumor cells may be subject to NMD inhibition. On one hand, the tumor microenvironment may inhibit NMD [5]. For example, the tumor cells often undergo cellular stresses, such as amino acid deprivation, hypoxia, and reactive oxygen species (ROS) generation. These stresses can cause the phosphorylation of the translation initiation factor *eIF2α* (short for α subunit of eukaryotic initiation factor 2), which in turn suppresses NMD [5–7]. On the other hand, the mutations in NMD effectors may inactivate NMD. For example, NMD effector *upf1* is frequently mutated in pancreatic adenosquamous carcinoma [8]. If NMD is strongly inhibited in cancers, then further inhibition of NMD would not express many new antigens and in turn no strong immune reactions.

In this study, we develop three metrics to measure NMD activities and use them to assess NMD activities in the samples of lung adenocarcinoma -- the most common histological type of lung cancers.

## Methods

### Data collection

We downloaded RNA-sequencing reads of lung adenocarcinoma patients from the NCBI Gene Expression Omnibus (GEO) database (accession number GSE40419) [9]. Only the data of 72 individuals with both tumor and adjacent normal tissues (i.e., 144 samples) were extracted and used in the study. The ages of the patients vary from 38 to 82 years old.

### Processing of RNA-seq data

Raw fastQ-formatted sequence files were mapped onto human reference genome (hg19) by using Tophat v2.0.8b [10], with annotated transcripts from Ensembl 71 [11] as a guide for mapping (using the option -G). After mapping, the expression of genes was estimated using Cufflinks v2.1.1 [12] and expressed as FPKM (Fragments per kilobase of transcript per million mapped reads). Extremely low expressed genes (less than ten reads in half or more of 144 samples) were excluded. We then normalized the data using the 75% percentile of each sample.

Afterwards, we applied samtools v1.1 [13] to identify candidate variants that exist in both tumor and normal samples for each individual by feeding both mapped reads files. To reduce the chance of regarding sequencing errors as single-nucleotide variations (SNVs), we extracted SNVs with the following criteria: 1) ≥ 5 reads covering a site in both tissues, and 2) both reference and variant alleles were supported by mapped reads. SNPeff [14] was then used to evaluate the predicted effect of each variant based on NCBI Refseq annotation. The output contained information of whether a variant can introduce PTCs and trigger NMD.

### Identifying NMD sensitive and insensitive genes

We compiled NMD-affected genes from four studies [15–18] in order to reliably define NMD target and non-target genes. Genes that are not included or not expressed in any of the four studies were excluded to avoid background biases. Specifically, we required that selected genes: i) had probe information in the two array-based studies [15, 18]; ii) met Hidenori Tani et al. standards [16], and iii) had at least one transcript isoform with ≥1 FPKM upon UPF1 knockdown in reference [17]. The filtering resulted in 8319 genes.

Then genes were classified into NMD targets if they met either of the criteria: i) ≥ 2-fold upregulation upon Upf1 knockdown according to references [15, 16, 18]; ii) having at least one transcript isoform upregulated ≥ 3-fold upon Upf1 knockdown and expressed ≥ 5 FPKM according to reference [17]. Finally, we obtained 817, 82, 37, and 13 target genes, depending on the number of supporting studies, 1, 2, 3, and 4 studies, respectively. The other genes that have no or marginal up-regulation (i.e., < 1.5-folds up-regulated in [15, 18], and < 2-folds up-regulated in [17]) and not stabilized according to [16] were classified as NMD non-target genes.

### Identifying NMD-specific exon skipping events

Theoretically, any alternative splicing events introducing PTCs may trigger NMD. For simplicity, here we considered only exon-skipping events. We also required that the upstream and downstream exons of a focused exon are not subject to alternative splicing to ensure that an NMD isoform is generated by alternatively splicing of the focused exon only. In this way, we obtained 776 exon-skipping events that may trigger NMD according to the 50/55-bp rule [19].

When calculating the expression levels of NMD-inducing and NMD-free splicing isoforms, we only use the following mapped reads to ensure accuracy: 1) mapping quality = 50; 2) covering at least 6 nts on each of the joined exons, and 3) no mismatches or indels within the 12 nts near exon-exon junctions. Then the expression levels of different isoforms were calculated by counting supporting reads. For metric design, only splicing events with more than ten supporting reads in both tumor and normal samples of a patient were used.

## Results

We use the following three metrics to measure NMD activity in a biological sample: (1) the mRNA expression level of NMD target genes, (2) the usage (i.e., percentage) of NMD-inducing splicing isoform in NMD target genes which have both NMD-inducing and NMD-free isoforms, and (3) the abundance ratio of mRNAs derived from the NMD-inducing allele to the NMD-free one of the same gene. We name these three metrics as *R*_*mRNA*_, *R*_*isoform*_, and *R*_*allele*_, respectively. In principle, if NMD activity is strong in a sample, these metrics will have small values, because the NMD-inducing forms are more effectively degraded. To calculate these metrics, we collected RNA-seq data from a large-scale study [9] which produced data for 72 patients with each patient having both tumor and adjacent normal tissue samples sequenced (Additional file 1: Table S1).

**Fig. 1:**
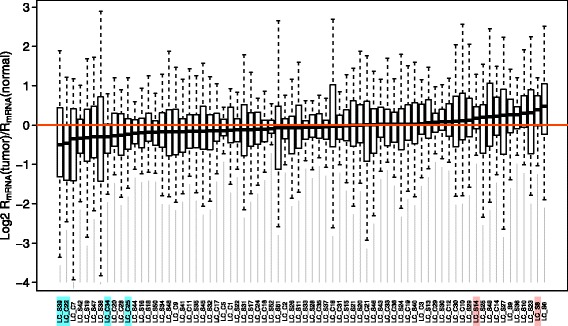
Distribution of *R*_*mRNA*_(*tumor*)/*R*_*mRNA*_(*normal*) among lung adenocarcinoma patients. Patients with significantly lower or higher *R*_*mRNA*_ in tumor than adjacent normal tissues (FDR, or false discovery rate, adjusted paired Wilcoxon rank sum test *P* value < 0.05) are highlighted with cyan or red, respectively

We first describe the procedure to calculate the metric *R*_*mRNA*_, i.e., the mRNA abundances of NMD target genes. The first step was to identify NMD target genes. For that, we collected NMD target genes from four studies [15–18]. These studies measured gene expression changes after inhibiting NMD. We classify all genes whose expression was upregulated upon NMD inhibition as NMD targets. Note the targets by this approach may include direct as well as indirect NMD targets, but our purpose is to identify genes which can be used as an indicator of NMD activity, so any genes with upregulated expression after NMD inhibition are informative. In total, 951 genes are classified as NMD targets by at least one study, and 50 genes are supported by at least three studies (see Methods section). For accuracy, we use these 50 genes as our NMD target gene set (Additional file 2). To eliminate gene expression variation across samples due to systematic bias, we normalize the expression of NMD targets by dividing it with the median expression value of the 4074 non-target genes (Additional file 2; also see Methods). Then *R*_*mRNA*_ is calculated for each gene using the following Eq. (1):

1 $$ {R}_{mRNA}={mE}_{NMD}/ median\_{mE}_{nonNMD} $$

where *mE*_*NMD*_ is the mRNA expression of an NMD target gene and *median*_*mE*_*nonNMD*_ is the median value of all 4074 non-target genes from the same sample. To infer NMD activity in tumor relative to in a normal tissue, *R*_*mRNA*_(*tumor*)/*R*_*mRNA*_(*normal*) is calculated, i.e., the ratio of *R*_*mRNA*_ between a tumor and the corresponding normal tissue. As shown in Fig. 1, the ratio *R*_*mRNA*_(*tumor*)/*R*_*mRNA*_(*normal*) varies both among genes and among patients, and in a few patients it deviates from unity significantly (Fig. 1; Additional file 1: Table S2). This result suggests that most tumor samples have NMD activity comparable to their normal baselines and that some tumor samples experienced dramatic changes in NMD activity.

**Fig. 2:**
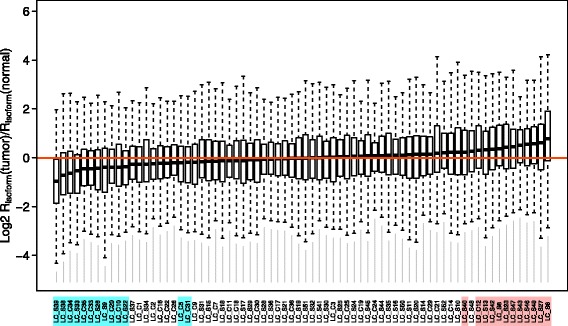
Distribution of *R*_*isoform*_(*tumor*)/*R*_*isoform*_(*normal*) among patients. Patients with significantly lower or higher *R*_*isoform*_ in tumor than adjacent normal tissues (FDR adjusted paired Wilcoxon rank sum test *P* value < 0.05) are highlighted with cyan or red, respectively

Next, we calculated *R*_*isoform*_, the usage of NMD-inducing alternative splicing (AS) isoforms in a given gene, as follows:

2 $$ {R}_{isoform}={sE}_{NMD}/\left({sE}_{NMD}+{sE}_{nonNMD}\right) $$

where *sE*_*NMD*_ and *sE*_*nonNMD*_ are the abundances of NMD-inducing and NMD-free splicing isoforms, respectively. Compared to the metric *R*_*mRNA*_, *R*_*isoform*_ is supposed to be more sensitive for two reasons: (1) it can detect NMD activity changes when the changes only affect the relative abundance of NMD-inducing splicing isoform but not the total mRNA abundance of a gene; (2) *R*_*isoform*_ uses the abundance of NMD-free isoforms of the same gene as a normalizing factor which is better than using the expression of other genes as this factor (as in *R*_*mRNA*_), because it is possible that the expression dynamics across samples may vary among genes. For calculating *R*_*isoform*_, we identified 776 AS events from 734 genes. Five hundred and twenty-eight and 248 cases trigger NMD when the alternative exon is included and excluded, respectively (see Methods for details). Among them, 14 AS events occur in all the samples (Additional file 3). To calculate *R*_*isoform*_ in each sample, we use either all informative AS events occurring in the sample or only the 14 shared AS events. Similar to calculating *R*_*mRNA*_, we use the ratio of *R*_*isoform*_(tumor) to *R*_*isoform*_(normal) to account for the baseline difference among patients. As shown in Fig. 2 and Additional file 1: Table S3, when using all informative AS events in each sample, the ratio *R*_*isoform*_(tumor)/*R*_*isoform*_(normal) significantly deviates from unity in dozens of patients. The number of significant deviations is much larger than that when using *R*_*mRNA*_, verifying the inference of higher sensitivity by *R*_*isoform*_. When using the 14 shared AS events, we basically observe the same pattern (Additional file 1: Figure S1, Spearman’s Rho = 0.6127131, *P* = 1.063e-08). Although the metric *R*_*isoform*_ is more sensitive than *R*_*mRNA*_, the ratios of tumor to normal samples from the two metrics are positively correlated (Additional file 1: Figure S2A, Rho = 0.3690621, *P* = 0.001422). The relationship remains when using the 14 shared events only (Additional file 1: Figure S2B, Rho = 0.2581838, *P* = 0.02882).

**Fig. 3:**
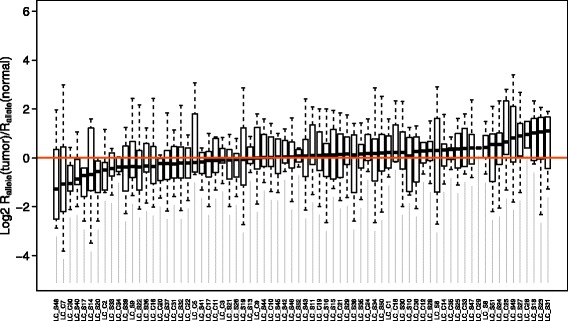
Distribution of *R*_*allele*_(*tumor*)/*R*_*allele*_(*normal*) among patients. In no patient *R*_*allele*_ shows significant difference between tumor and adjacent normal tissues

Finally, we calculate *R*_*allele*_ as follows:

$$ {R}_{allele}={aE}_{NMD}/{aE}_{nonNMD}, $$

where *aE*_*NMD*_ and *aE*_*nonNMD*_ are the abundances of mRNAs derived from the NMD-inducing and NMD-free alleles of the same gene, respectively. For this analysis, all detected heterozygous nonsense mutations are included (Additional file 4). And again, the ratio of *R*_*allele*_(tumor) to *R*_*allele*_(normal) is calculated for each patient to infer NMD activity change. This approach has been used in multiple studies to check NMD efficiency [20–22]. However, in our analysis we do not detect significant difference between tumor and normal samples for any patient, probably due to a limited number of sites (Fig. 3; Additional file 1: Table S4). Nevertheless, a positive correlation between the median ratios of *R*_*allele*_(*tumor*)/*R*_*allele*_(*normal*) and *R*_*mRNA*_(*tumor*)/*R*_*mRNA*_(*normal*) across patients is observed (Additional file 1: Figure S3A, Rho = 0.2134399, *P* = 0.07183). And similarly, a slightly better correlation between (*tumor*)/*R*_*allele*_(*normal*) and *R*_*isoform*_(*tumor*)/*R*_*isoform*_(*normal*) is observed (Additional file 1: Figure S3B, Rho = 0.3099928, *P* = 0.00805). These results indicate that the metric *R*_*allele*_ can capture the NMD activity change in tumors, but more sites are needed for making the metric effective enough.

Next we examine how these metrics are correlated with the expression of nine NMD effectors, including *Upf1, Upf2, Upf3a, Upf3b, Smg1, Smg5, Smg6, Smg7* and *Pnrc2* [23, 24]. Theoretically, we expect a negative correlation between the metrics of NMD activity and the expression of these factors, because a smaller metric value means stronger NMD activity conferred by higher expression of NMD effectors. We found that *R*_*mRNA*_ is negatively correlated with the expression of *smg1, smg7, upf2* and *pnrc2,* though not always statistically significant (Additional file 1: Table S5). Surprisingly, *R*_*mRNA*_ is positively correlated with the expression of *upf1*, *upf3a*, and *smg6* in some cases. These results suggest that NMD effectors may have contributed to NMD activity in a discordant way, with some effectors being more important than other in affecting NMD activity. However, it is unclear why some effectors such as upf1 are expressed at higher level where the NMD activity appears weaker. We also examine similar correlations by using *R*_*isoform*_, and none of these is significant and the signs of correlation are not always consistent with expectation (Additional file 1: Table S5). These together suggest that the change of NMD activity may not be consistent with the expression change of all NMD effectors, and thus a better understanding of NMD effectors in a regulatory network is needed for inferring NMD activity from the effectors’ expression.

## Discussion

A key step for applying NMD-inhibition based therapies is to measure NMD activity accurately. In this study, we try to reach this goal from three aspects. (1) We use the expression of many NMD target genes to infer NMD activity, because using a few genes may be less sensitive in detecting NMD activity change as the NMD-targeting efficiency varies among genes [25] and conditions, so using many genes imparts a better statistical power. (2) We develop three metrics to corroborate each other. Indeed, the three metrics are moderately correlated. Particularly, both *R*_*mRNA*_ and *R*_*isoform*_ show consistent and significant results in a few patients, such as LC_S39, LC_C34 and LC_S8. (3) We use the metric value in respective normal tissues to normalize that in tumors, through which we can eliminate biases introduced by factors that affect the expression in both normal and tumor samples (but also see next paragraph). For example, if an NMD-inducing allele is expressed lower than an NMD-free one in both normal and tumor samples due to associated polymorphic *cis-*regulatory elements, then normalization would eliminate this effect and avoid overestimating NMD activity in this case.

Despite our efforts, the method can be further improved in future. First, the NMD target gene set can be refined to improve sensitivity. We compiled the gene set based on mRNA expression change upon NMD inhibition. The set may include both direct and indirect NMD targets. Indirect targets however may not change their expression consistently when NMD activity changes, because their expression may be mainly regulated by other pathways rather than by NMD. Using only direct NMD targets may improve the accuracy of the method. Second, other NMD-inducing features may also be used to measure NMD activity. In our design, we use either the total mRNA expression of NMD target genes or the mRNA isoforms derived from alternative splicing or nonsense mutation alleles to monitor NMD activities. Theoretically, the presence of a uORF and the length of 3’UTR can also be used to select NMD target mRNAs and thus use their (relative) expression to monitor NMD activities. However, these features may not be as robust as the 50/55-bp rule (used for selecting NMD-targeted isoforms in our study), such as only translated uORFs can trigger NMD [26]. Therefore, incorporating these features need further testing. Third, we assume that the NMD activity varies little in normal samples and use it to normalize that in tumors. This assumption may not be absolutely true and normalization may over- or under- estimate the true NMD activity in a tumor sample. It is therefore worthwhile to compare the performances of the metrics with and without normalization.

A good approach of measuring NMD activity is valuable for clinical applications as NMD influences a variety of physiological and pathological processes. For example, the high mutation rate of *Upf1* in pancreatic adenosquamous carcinoma [8] and its downregulation in hepatocellular carcinoma [27] indicate that the NMD pathway could be frequently suppressed. Therefore, an estimate of NMD activity can guide NMD-based therapies. In lung adenocarcinoma, we find that the NMD activity can either decrease or increase compared to adjacent normal tissues, suggesting that NMD-inhibition based therapies may result in better effects in some patients than others. Furthermore, we don’t observe any correlation between NMD activity and tumor regression (Additional file 1: Table S6, *P* > 0.14), suggesting that NMD-inhibition based approach may be applicable to tumors of any stage. Actually, an estimate of NMD activity is also informative for diseases other than tumors.

For instance, “PTC read through” drugs have been used to restore the translation of PTC-containing transcripts for diseases such as cystic fibrosis and Duchenne muscular dystrophy etc. in several pilot clinical trials [28]. An estimate of NMD activity in patients can therefore help personalized medicine.

## Conclusions

We developed three metrics for inferring NMD activities based on RNA-seq data. Among them, the metric *R*_*isoform*_ performs the best due to a moderate size of used target genes and using NMD-free splicing isoforms as a natural control. Our results suggest that NMD activity varies among patients and that the metrics may be used to assess NMD activities in other types of diseases.

## Additional files

Additional file 1: This document contains all supplementary Tables and supplementary Figures with legends. (PDF 473 kb)

Additional file 2: This document contains information of NMD target and non-target genes among patients. (TXT 4665 kb)

Additional file 3: This document contains information of the alternative exons that can distinguish NMD target and non-target isoforms among patients. (TXT 845 kb)

Additional file 4: This document contains information of the PTC inducing SNVs among patients. (TXT 25 kb)

## Abbreviations

eIF2 α: α subunit of eukaryotic initiation factor 2
EJC: Exon junction complex
FPKM: Fragments per kilobase of transcript per million mapped reads
GEO: Gene Expression Omnibus
NMD: Nonsense-mediated mRNA decay
PTC: Premature termination codon
RNA-seq: Whole transcriptome high-throughput sequencing
ROS: Reactive oxygen species
