# Supplementary material for: Assessing the activity of nonsense-mediated mRNA decay in lung cancer
Source: BMC Med Genomics. 2017 Sep 6;10:55. doi: 10.1186/s12920-017-0292-z (PMC5586017; doi:10.1186/s12920-017-0292-z)
Supplement: Supplementary file 1 — This document contains all supplementary Tables and supplementary Figures with legends. (PDF 473 kb) [file 12920_2017_292_MOESM1_ESM.pdf]

Supplementary materials for Wang, et al, Additional file 1

Supplementary materials for Wang, et al, Additional file 1 1
Supplementary Tables 2
Supplementary Figures 13

Supplementary Tables

Table S1. Sample information.

Patient label	Age at diagnosis	Gender (1=male, 2=female)	Smoking_status (0=neverSmoker; 1=smoker; 2=current_smoker ; 3=unknown)	Tumor stage	Lymph node metastasis (0=No, 1=Yes)	Tumor accession	Normal accession	Tumor reads	Normal reads
LC_C1	54	1	1	1A	0	ERR164550	ERR164473	38747243	22844380
LC_C10	58	2	0	2A	1	ERR164559	ERR164479	45442873	28952003
LC_C11	63	2	0	1A	0	ERR164560	ERR164480	36345502	30034560
LC_C12	66	1	3	1B	0	ERR164561	ERR164481	33751833	27199448
LC_C14	50	2	2	3A	1	ERR164563	ERR164482	39577218	33297988
LC_C16	54	2	0	1A	0	ERR164565	ERR164483	38599387	30379431
LC_C17	64	2	0	1A	0	ERR164566	ERR164484	37570412	33270003
LC_C18	68	2	1	1B	0	ERR164567	ERR164485	43603998	36204547
LC_C19	40	2	0	4	0	ERR164568	ERR164486	39607756	37810104
LC_C2	51	1	2	2B	0	ERR164551	ERR164474	53607774	42022150
LC_C20	65	1	1	1A	0	ERR164569	ERR164487	36620521	34810994
LC_C21	42	1	1	1B	0	ERR164570	ERR164488	33031428	38877376
LC_C22	73	1	1	1A	0	ERR164571	ERR164489	36827801	29768181
LC_C24	66	2	0	1A	0	ERR164573	ERR164490	34446009	30552607
LC_C25	66	1	1	1A	0	ERR164574	ERR164491	40862036	40061444
LC_C28	59	2	0	NA	0	ERR164577	ERR164492	52965117	31118616
LC_C29	60	2	0	4	0	ERR164578	ERR164493	52251114	30267534
LC_C3	65	2	0	1A	0	ERR164552	ERR164475	41576054	36558196
LC_C30	38	2	0	1A	0	ERR164579	ERR164494	50680901	32973203
LC_C31	61	2	0	1A	0	ERR164580	ERR164495	53023203	29426650
LC_C33	62	1	3	NA	0	ERR164582	ERR164496	56634311	27604788

LC_C34	73	1	1	1B	0	ERR164583	ERR164497	49876146	25731489
LC_C35	60	1	2	2A	1	ERR164584	ERR164498	50381230	31485881
LC_C36	56	2	0	1A	0	ERR164585	ERR164499	41887225	28180652
LC_C5	68	1	3	3A	1	ERR164554	ERR164476	39009132	32065580
LC_C7	81	1	1	1A	0	ERR164556	ERR164477	45536591	35823521
LC_C9	71	1	1	1B	0	ERR164558	ERR164478	37259703	42992272
LC_S10	69	1	1	4	1	ERR164590	ERR164508	25639079	36929327
LC_S11	65	1	1	1B	0	ERR164591	ERR164509	40642669	44219885
LC_S13	62	1	1	1B	0	ERR164593	ERR164510	39817881	31899940
LC_S14	79	1	1	1B	0	ERR164594	ERR164511	35208393	31290309
LC_S15	73	1	2	1B	0	ERR164595	ERR164512	55195368	39920425
LC_S16	67	2	0	1A	0	ERR164597, ERR164596	ERR164513	45149896	36408281
LC_S17	73	1	1	1A	0	ERR164599, ERR164598	ERR164514	52331896	35726514
LC_S18	76	1	1	3A	1	ERR164600	ERR164515	96081958	31497329
LC_S19	62	1	1	1A	0	ERR164601	ERR164516	60414304	34642008
LC_S20	58	1	1	1B	0	ERR164602	ERR164517	57662415	31193217
LC_S21	52	1	2	1A	0	ERR164603	ERR164518	63856760	36693314
LC_S22	67	2	0	1B	0	ERR164604	ERR164519	52237502	34871202
LC_S23	62	2	0	1B	0	ERR164605	ERR164520	55302491	35109105
LC_S24	70	2	0	2A	1	ERR164606	ERR164521	65138060	36070935
LC_S25	78	1	1	3A	1	ERR164607	ERR164522	61863976	37385715
LC_S26	70	2	0	1B	0	ERR164608	ERR164523	60720273	33943554
LC_S27	65	1	1	1A	0	ERR164609	ERR164524	57606955	29128935
LC_S28	63	1	0	1B	0	ERR164610	ERR164525	54933146	37843199
LC_S29	59	1	2	3A	1	ERR164611	ERR164526	63317174	31527488
LC_S30	66	2	0	1B	0	ERR164612	ERR164527	55724222	33656997
LC_S31	56	1	1	2B	0	ERR164613	ERR164528	67129037	38815656
LC_S32	64	1	1	1A	0	ERR164614	ERR164529	57751163	39369979

LC_S33	66	2	0	1A	0	ERR164615	ERR164530	63332457	28568100
LC_S34	45	2	0	1B	0	ERR164616	ERR164531	61887903	33008257
LC_S35	82	1	1	4	1	ERR164617	ERR164532	54159676	34867292
LC_S36	74	2	0	1B	0	ERR164618	ERR164533	49749450	38983377
LC_S37	69	1	1	2B	0	ERR164619	ERR164534	67385676	35282174
LC_S38	65	1	2	3A	1	ERR164620	ERR164535	60253340	17270628
LC_S39	58	1	1	2B	1	ERR164621	ERR164536	66588273	38797818
LC_S40	47	1	2	2B	0	ERR164622	ERR164537	33747468	28086427
LC_S41	55	2	1	3A	1	ERR164623	ERR164538	34223354	35274948
LC_S42	62	2	0	3B	1	ERR164624	ERR164539	41045273	52034997
LC_S43	68	2	0	1B	0	ERR164625	ERR164540	34893898	47760113
LC_S44	75	2	0	1A	0	ERR164626	ERR164541	34779422	46858874
LC_S45	72	1	0	1A	0	ERR164627	ERR164542	37334315	50435154
LC_S46	66	1	0	1B	0	ERR164628	ERR164543	36607936	43998403
LC_S47	75	2	0	3B	0	ERR164629	ERR164544	32000887	45519810
LC_S48	48	2	0	1B	0	ERR164630	ERR164545	32015184	52624636
LC_S49	66	2	0	1A	0	ERR164631	ERR164546	35692599	38877419
LC_S50	64	2	0	1B	0	ERR164632	ERR164547	35163857	37039026
LC_S51	64	1	2	2A	0	ERR164633	ERR164548	38627258	39530504
LC_S52	82	2	1	1A	0	ERR164634	ERR164549	33115066	46480356
LC_S6	58	1	1	1A	0	ERR164587, ERR164586	ERR164505	82913497	45440332
LC_S8	71	2	0	1A	0	ERR164588	ERR164506	22541432	41766400
LC_S9	69	1	1	2B	0	ERR164589	ERR164507	1.09E+08	36620147

Table S2. R_{mRNA} and their ratio between tumor and normal samples.

Patient label	Median R_{mRNA}(tumor)	Median R_{mRNA}(normal)	Median R_{mRNA}(tumor)/ R_{mRNA}(normal)	P value	FDR
LC_S39	0.868	1.257	0.710	0.003	0.039
LC_C22	0.930	1.294	0.728	0.000	0.007
LC_C7	0.895	1.180	0.789	0.032	0.128
LC_S42	1.094	1.291	0.794	0.006	0.053
LC_S19	1.118	1.267	0.798	0.231	0.426
LC_S47	1.257	1.181	0.815	0.076	0.218
LC_S38	1.083	1.672	0.818	0.150	0.318
LC_C34	1.039	1.286	0.819	0.001	0.020
LC_C20	1.091	1.313	0.826	0.076	0.218
LC_C28	1.199	1.259	0.840	0.007	0.053
LC_C25	1.117	1.293	0.850	0.003	0.039
LC_S44	1.170	1.263	0.868	0.010	0.071
LC_S16	1.152	1.264	0.877	0.011	0.071
LC_S18	1.294	1.168	0.879	0.315	0.505
LC_S50	1.077	1.256	0.881	0.019	0.108
LC_S34	1.048	1.257	0.889	0.213	0.415
LC_S48	1.127	1.158	0.892	0.180	0.370
LC_C9	0.982	1.021	0.893	0.237	0.426
LC_S41	0.976	1.175	0.893	0.028	0.128
LC_C11	1.105	1.236	0.896	0.297	0.486
LC_S35	1.179	1.220	0.897	0.118	0.279
LC_S45	1.219	1.210	0.897	0.499	0.666
LC_S32	1.231	1.144	0.904	0.385	0.554
LC_C17	1.110	1.193	0.906	0.339	0.520
LC_C5	1.132	1.218	0.908	0.023	0.120
LC_C1	1.128	1.204	0.916	0.802	0.875
LC_S22	1.150	1.244	0.920	0.026	0.124
LC_S31	1.018	1.098	0.925	0.247	0.433
LC_S17	1.157	1.191	0.925	0.125	0.281
LC_C24	1.263	1.285	0.933	0.369	0.550
LC_C16	1.061	1.237	0.934	0.113	0.279
LC_S52	1.124	1.102	0.939	0.065	0.218
LC_S51	0.881	1.056	0.953	0.334	0.520

LC_C2	1.000	1.001	0.954	0.042	0.158
LC_S26	0.984	1.213	0.958	0.073	0.218
LC_S11	1.147	1.086	0.959	0.699	0.799
LC_S33	1.167	1.255	0.965	0.199	0.398
LC_S28	1.141	1.168	0.967	0.284	0.475
LC_C35	1.153	1.193	0.967	0.499	0.666
LC_S37	1.029	1.022	0.973	0.088	0.242
LC_C18	1.139	1.036	0.981	0.650	0.755
LC_C31	1.037	1.169	0.982	0.643	0.755
LC_S15	1.162	1.172	0.987	0.772	0.855
LC_S21	1.042	1.189	1.000	0.900	0.953
LC_S20	1.158	1.117	1.001	0.969	0.983
LC_C21	1.061	1.130	1.005	0.556	0.715
LC_S46	1.240	1.292	1.010	0.629	0.755
LC_S43	1.144	1.060	1.014	0.969	0.983
LC_C33	1.143	1.131	1.014	0.650	0.755
LC_C36	1.270	1.193	1.014	0.877	0.943
LC_S24	1.246	1.117	1.016	0.401	0.566
LC_C19	1.130	1.180	1.020	0.992	0.992
LC_S40	0.990	1.087	1.020	0.728	0.819
LC_C3	1.332	1.071	1.026	0.616	0.755
LC_S13	1.341	1.201	1.040	0.417	0.578
LC_C29	1.118	1.060	1.051	0.142	0.310
LC_S30	1.323	1.205	1.060	0.374	0.550
LC_C12	1.330	1.094	1.063	0.231	0.426
LC_C30	1.266	1.233	1.072	0.543	0.711
LC_C10	1.384	1.070	1.083	0.284	0.475
LC_S29	1.162	1.100	1.094	0.589	0.744
LC_S14	1.305	1.146	1.130	0.003	0.039
LC_S25	0.997	1.216	1.152	0.915	0.955
LC_S49	1.678	1.346	1.157	0.120	0.279
LC_C14	1.318	1.143	1.167	0.076	0.218
LC_S27	1.901	1.264	1.194	0.118	0.279
LC_S9	1.255	1.299	1.201	0.056	0.201
LC_S36	1.318	1.054	1.201	0.006	0.053
LC_S10	1.550	1.263	1.231	0.017	0.100
LC_S23	1.390	1.174	1.241	0.107	0.279
LC_S8	1.827	1.305	1.311	0.000	0.012

LC_S6	2.234	1.423	1.401	0.031	0.128

Table S3. $R_{isoform}$ and their ratio between tumor and normal samples

Patient label	Median $R_{isoform}$(tumor)	Median $R_{isoform}$(normal)	Median $R_{isoform}$(tumor)/ $R_{isoform}$(normal)	P value	FDR
LC_S39	0.026	0.053	0.515	0.000	0.000
LC_S38	0.042	0.053	0.605	0.002	0.007
LC_C34	0.031	0.052	0.642	0.000	0.001
LC_S33	0.037	0.059	0.693	0.000	0.000
LC_C35	0.034	0.050	0.728	0.000	0.000
LC_C33	0.034	0.043	0.733	0.000	0.001
LC_S26	0.034	0.050	0.744	0.000	0.001
LC_S9	0.042	0.052	0.762	0.001	0.005
LC_C20	0.043	0.053	0.768	0.002	0.007
LC_C10	0.042	0.053	0.769	0.008	0.025
LC_S22	0.040	0.051	0.770	0.000	0.000
LC_S37	0.035	0.040	0.828	0.022	0.060
LC_C1	0.040	0.051	0.833	0.049	0.122
LC_S34	0.044	0.039	0.837	0.062	0.148
LC_C2	0.035	0.043	0.846	0.048	0.122
LC_C16	0.047	0.048	0.855	0.117	0.235
LC_C22	0.047	0.047	0.862	0.186	0.334
LC_C28	0.048	0.050	0.865	0.274	0.420
LC_C5	0.039	0.043	0.865	0.008	0.025
LC_C31	0.040	0.053	0.881	0.002	0.008
LC_C9	0.041	0.047	0.884	0.047	0.122
LC_S31	0.036	0.043	0.890	0.115	0.235
LC_S15	0.039	0.045	0.911	0.877	0.929
LC_C7	0.038	0.042	0.912	0.623	0.724
LC_S18	0.038	0.042	0.914	0.417	0.556
LC_C11	0.051	0.062	0.914	0.127	0.247
LC_C18	0.038	0.044	0.915	0.138	0.260
LC_S17	0.052	0.055	0.920	0.432	0.565
LC_S29	0.039	0.042	0.950	0.393	0.534
LC_C30	0.048	0.056	0.950	0.840	0.916

LC_S28	0.039	0.044	0.957	0.312	0.432
LC_S36	0.047	0.052	0.961	0.941	0.954
LC_C17	0.048	0.048	0.977	0.601	0.721
LC_S21	0.042	0.046	0.979	0.479	0.594
LC_C36	0.043	0.044	0.980	0.932	0.954
LC_S19	0.051	0.053	0.985	0.615	0.724
LC_S51	0.043	0.037	0.993	0.472	0.594
LC_S32	0.037	0.036	0.997	0.977	0.977
LC_S41	0.042	0.038	1.000	0.311	0.432
LC_S30	0.044	0.051	1.014	0.912	0.952
LC_C3	0.040	0.041	1.016	0.724	0.815
LC_S25	0.043	0.039	1.027	0.574	0.701
LC_C25	0.050	0.056	1.028	0.761	0.843
LC_S24	0.034	0.038	1.047	0.293	0.422
LC_C19	0.048	0.046	1.047	0.856	0.919
LC_S45	0.042	0.036	1.052	0.211	0.361
LC_C24	0.058	0.054	1.054	0.083	0.175
LC_S44	0.048	0.046	1.057	0.256	0.410
LC_S35	0.037	0.040	1.072	0.216	0.361
LC_S16	0.046	0.047	1.075	0.683	0.780
LC_S50	0.047	0.043	1.078	0.227	0.372
LC_S11	0.040	0.043	1.087	0.293	0.422
LC_S20	0.043	0.043	1.100	0.212	0.361
LC_C29	0.055	0.043	1.105	0.288	0.422
LC_S14	0.046	0.043	1.105	0.470	0.594
LC_C21	0.050	0.039	1.132	0.141	0.260
LC_S52	0.050	0.047	1.154	0.064	0.150
LC_C14	0.043	0.042	1.163	0.272	0.420
LC_S10	0.051	0.046	1.164	0.071	0.159
LC_S40	0.054	0.043	1.177	0.010	0.029
LC_S48	0.039	0.032	1.211	0.079	0.172
LC_C12	0.058	0.047	1.233	0.000	0.002
LC_S13	0.066	0.048	1.264	0.001	0.005
LC_S42	0.051	0.046	1.273	0.014	0.041
LC_S8	0.080	0.057	1.293	0.000	0.000
LC_S23	0.062	0.051	1.354	0.000	0.002
LC_S47	0.051	0.036	1.376	0.000	0.001
LC_S43	0.063	0.046	1.419	0.000	0.000

LC_S46	0.065	0.044	1.465	0.000	0.000
LC_S49	0.074	0.046	1.488	0.000	0.002
LC_S27	0.062	0.040	1.527	0.000	0.001
LC_S6	0.097	0.047	1.712	0.000	0.000

Table S4. R_{allele} and their ratio between tumor and normal samples

Patient label	Median R_{allele}(tumor)	Median R_{allele}(normal)	Median R_{allele}(tumor)/ R_{allele}(normal)	P value	FDR
LC_C7	0.326	0.847	0.48	0.394	1
LC_C30	0.321	0.714	0.485	0.096	1
LC_S40	0.538	0.583	0.548	0.534	1
LC_S17	0.4	0.333	0.603	0.377	1
LC_S14	0.31	0.536	0.621	0.395	1
LC_S20	0.66	0.833	0.688	0.197	1
LC_C2	0.429	0.833	0.707	0.226	1
LC_S33	0.581	0.742	0.742	0.544	1
LC_C34	0.314	0.374	0.779	0.63	1
LC_S39	1.66	2.125	0.779	0.686	1
LC_S9	0.756	0.69	0.785	1	1
LC_S22	0.55	0.798	0.793	0.47	1
LC_S36	0.683	0.714	0.794	0.681	1
LC_C16	0.458	0.348	0.802	1	1
LC_C20	0.571	0.764	0.848	0.45	1
LC_S37	0.707	0.804	0.853	0.448	1
LC_C31	0.548	0.775	0.856	0.408	1
LC_S32	0.462	0.6	0.868	0.96	1

LC_C22	0.561	0.722	0.87	0.809	1
LC_C5	0.603	0.651	0.896	0.521	1
LC_S41	0.909	0.895	0.896	1	1
LC_C11	0.697	0.545	0.929	0.916	1
LC_C17	0.5	0.583	0.929	0.369	1
LC_C3	0.4	0.467	0.938	1	1
LC_S21	0.719	0.89	0.941	0.583	1
LC_S26	0.455	0.545	0.955	0.178	1
LC_S19	0.612	0.738	0.971	0.84	1
LC_S13	0.625	0.6	0.982	0.678	1
LC_C9	0.778	1	1	0.965	1
LC_S44	0.817	0.627	1.006	0.519	1
LC_C10	1.025	0.692	1.008	0.71	1
LC_S45	0.512	0.361	1.026	0.948	1
LC_S42	0.641	0.661	1.027	0.665	1
LC_S46	0.592	0.726	1.031	0.91	1
LC_S52	0.697	0.667	1.051	0.733	1
LC_S43	0.642	0.631	1.068	0.755	1
LC_S11	0.655	0.446	1.078	0.623	1
LC_C19	1.322	0.925	1.08	0.674	1
LC_S16	0.638	0.617	1.081	0.89	1
LC_S15	0.667	0.657	1.091	0.677	1
LC_C21	0.429	0.652	1.102	1	1
LC_S29	0.573	0.613	1.12	0.817	1
LC_S38	0.381	0.904	1.123	1	1
LC_S35	0.75	0.769	1.125	0.837	1
LC_C24	0.667	0.714	1.133	0.844	1
LC_S34	0.965	0.612	1.14	0.436	1

LC_S50	0.383	0.425	1.148	0.978	1
LC_C1	1.067	0.857	1.167	1	1
LC_C18	0.792	0.786	1.167	0.548	1
LC_S30	0.845	0.579	1.17	0.358	1
LC_S10	0.514	0.59	1.171	0.936	1
LC_C36	0.481	0.481	1.208	1	1
LC_C12	0.455	0.294	1.222	0.453	1
LC_S28	0.88	0.559	1.227	0.267	1
LC_S6	0.493	0.658	1.241	0.734	1
LC_C14	0.583	0.594	1.25	0.563	1
LC_C35	0.75	0.565	1.269	0.421	1
LC_S25	0.928	0.5	1.294	0.202	1
LC_C33	0.429	0.243	1.312	0.528	1
LC_C29	0.667	0.5	1.333	1	1
LC_S47	0.938	0.8	1.333	0.266	1
LC_S8	0.667	0.579	1.339	0.369	1
LC_S51	0.571	0.6	1.465	1	1
LC_S24	1.121	0.882	1.472	0.428	1
LC_C25	0.738	0.376	1.574	0.186	1
LC_S49	0.882	0.261	1.771	0.442	1
LC_S27	0.9	0.333	1.865	0.224	1
LC_C28	0.667	0.5	1.954	0.548	1
LC_S18	0.7	0.412	2.031	0.297	1
LC_S23	0.641	0.474	2.095	0.495	1
LC_S31	1.357	1	2.143	0.306	1

Table S5. a) Correlation between expression of NMD effectors and R_{mRNA} in both normal and tumor tissues; as well as their tumor/normal ratio with median R_{mRNA}(tumor)/R_{mRNA}(normal)

	R_{mRNA}(normal)		R_{mRNA}(tumor)		R_{mRNA}(tumor)/R_{mRNA}(normal)	
	Rho	P	Rho	P	Rho	P
upf1	-0.1857997	0.118	0.2117821	0.07421	0.272204	0.020993
upf2	0.2406586	0.04195	-0.0706476	0.5546	-0.23072	0.051391
upf3a	0.0802946	0.5017	0.2777027	0.01846	0.238504	0.043866
upf3b	0.0009325	0.9939	0.1839347	0.1218	0.103704	0.385161
smg1	0.2038395	0.08594	-0.2455785	0.03784	-0.3459386	0.003064
smg5	-0.0792977	0.507	0.04071001	0.7337	0.018458	0.877489
smg6	-0.1379188	0.2474	0.1535147	0.1975	0.294874	0.01218
smg7	0.0870474	0.4663	-0.1898514	0.1101	-0.21091	0.075427
pnrc2	-0.1308123	0.2727	-0.219886	0.06361	-0.18847	0.112768

b) Correlation between expression of NMD effectors and $R_{isoform}$ in both normal and tumor tissues; as well as their tumor/normal ratio with median $R_{isoform}$(tumor)/$R_{isoform}$(normal)

	$R_{isoform}$(normal)		$R_{isoform}$(tumor)		$R_{isoform}$(tumor)/$R_{isoform}$(normal)	
	Rho	P	Rho	P	Rho	P
upf1	-0.2127003	0.07284	0.1442387	0.2267	-0.02260614	0.8505
upf2	0.02566102	0.8306	0.05920042	0.6213	0.0446013	0.7099
upf3a	-0.03644958	0.7611	0.1812993	0.1275	0.1645939	0.1671
upf3b	-0.065777	0.583	0.2185849	0.06509	0.153741	0.1973
smg1	-0.299732	0.01053	0.03947231	0.742	0.2270743	0.05508
smg5	-0.105747	0.3767	0.06722351	0.5748	0.06936193	0.5626
smg6	0.2270904	0.05507	0.09249865	0.4396	-0.08471674	0.4792
smg7	0.156619	0.1889	-0.0879163	0.4627	-0.1264561	0.2898

pnrc2	-0.1458948	0.2214	-0.2313029	0.05059	-0.1565226	0.1892

Table S6. No association between NMD activity and tumor progression.

	Median R_{mRNA}(tumor)/ R_{mRNA}(normal)		Median $R_{isoform}$(tumor)/ $R_{isoform}$(normal)		Median R_{allele}(tumor)/ R_{allele}(normal)	
	Coefficient	P value	Coefficient	P value	Coefficient	P value
Tumor stage	0.00462	0.793	-0.0101	0.729	0.0656	0.142
Lymph node metastasis	-0.00698	0.862	-0.0936	0.159	0.0988	0.353

Supplementary Figures

Figure S1. $R_{isoform}(tumor)/R_{isoform}(normal)$ calculated from all informative events (>= 10 total supporting reads in both paired tissues of any patient) is significantly positively correlated (Spearman's Rho = 0.6127131, P = 1.063e-08) with that from the 14 shared events (>=10 supporting reads in all 144 samples).

Figure S2. $R_{mRNA}(tumor)/R_{mRNA}(normal)$ is significantly positively correlated with $R_{isoform}(tumor)/R_{isoform}(normal)$, regardless of whether all informative AS events (A, Rho = 0.3690621, P = 0.001422) or only the 14 shared events (B, Rho = 0.2581838, P = 0.02882) were used.

Figure S3. $R_{allele}(tumor)/R_{allele}(normal)$ is weakly positively correlated with $R_{mRNA}(tumor)/R_{mRNA}(normal)$ (A, Rho = 0.2134399, P = 0.07183), and strongly with $R_{isoform}(tumor)/R_{isoform}(normal)$ (B, Rho = 0.2581838, P = 0.02882).

(continue to next page)

Fig. S1

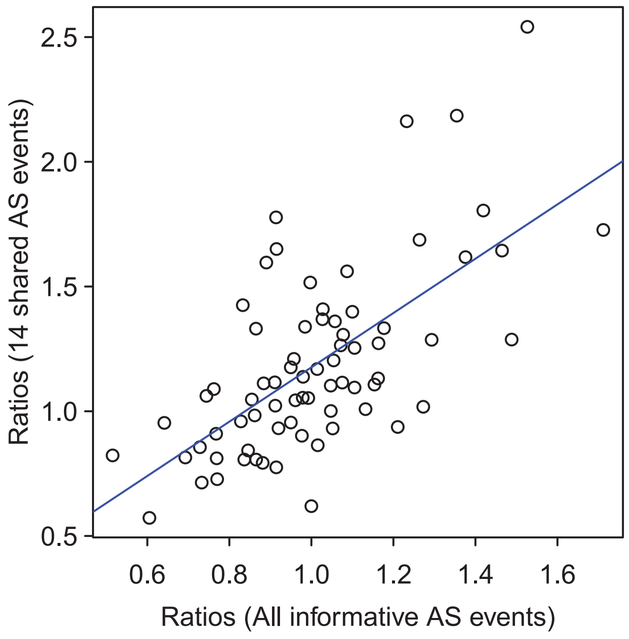
2.5
2.0
1.5
1.0
0.5
0.6
0.8
1.0
1.2
1.4
1.6
Ratios (14 shared AS events)
Ratios (All informative AS events)

Fig. S2

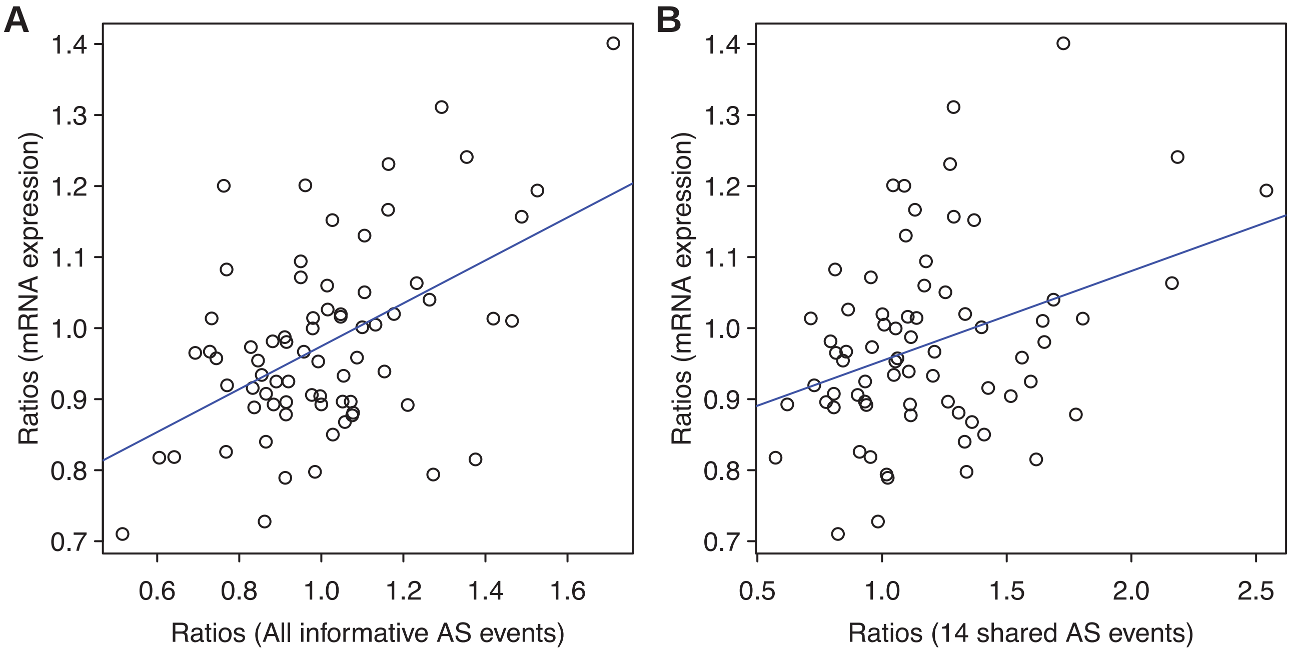
A
B
Ratios (mRNA expression)
1.4
1.3
1.2
1.1
1.0
0.9
0.8
0.7
0.6
0.8
1.0
1.2
1.4
1.6
Ratios (All informative AS events)
Ratios (mRNA expression)
0.5
1.0
1.5
2.0
2.5
Ratios (14 shared AS events)

Fig. S3

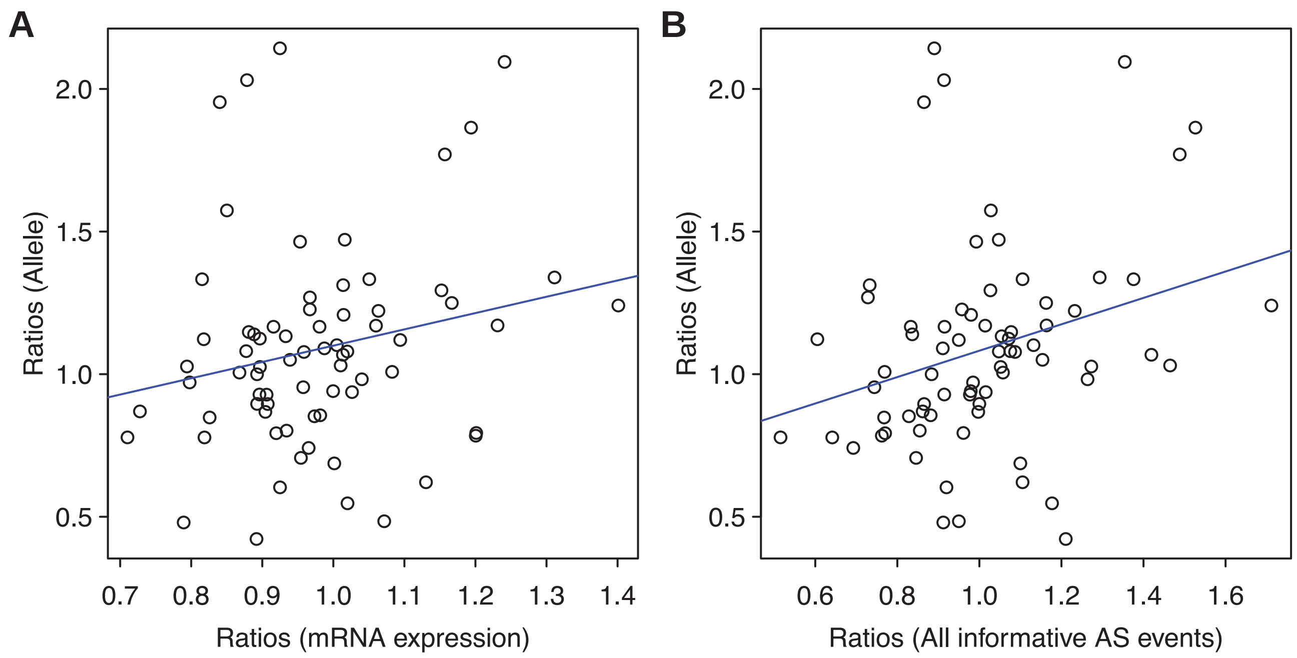
A
B
Ratios (Allele)
Ratios (mRNA expression)
Ratios (All informative AS events)
0.5
1.0
1.5
2.0
0.7
0.8
0.9
1.0
1.1
1.2
1.3
1.4
0.6
0.8
1.0
1.2
1.4
1.6
